# P-1123. Optimizing use of Cefazolin for Surgical Prophylaxis in Patients with Penicillin Allergy Labels

**DOI:** 10.1093/ofid/ofaf695.1318

**Published:** 2026-01-11

**Authors:** Amanda Binkley, Olajumoke O Fadugba, Patrick Kim, Laurel Adams, M Kit Delgado, Jeffrey Ebert, Athena Lee, Aria Xiong, Nikhil Mull, Kristel Frey, Arielle Berk, Jesal Shah, Naasha J Talati

**Affiliations:** Penn Presbyterian Medical Center, North Wales, PA; University of Pennsylvania, Philadelphia, Pennsylvania; Penn Presbyterian Medical Center, North Wales, PA; University of Pennsylvania, Philadelphia, Pennsylvania; University of Pennsylvania, Perelman School of Medicine, Philadelphia, Pennsylvania; Penn Medicine Nudge Unit, Philadelphia, Pennsylvania; University of Pennsylvania, Philadelphia, Pennsylvania; Penn Medicine Nudge Unit, Center for Health Care Transformation and Innovation, University of Pennsylvania Health System, Philadephia, Pennsylvania; University of Pennsylvania, Philadelphia, Pennsylvania; Penn Medicine, Philadelphia, Pennsylvania; University of Pennsylvania, Philadelphia, Pennsylvania; University of Pennsylvania, Philadelphia, Pennsylvania; Penn Presbyterian Medical Center, North Wales, PA

## Abstract

**Background:**

Cefazolin (CFZ), a common antibiotic used in surgical prophylaxis, is not always utilized when appropriate in patients with a penicillin (PCN) allergy due to concerns regarding potential cross-reactivity. Use of alternative antibiotics has been associated with increased risk of surgical site infections and delays in start of surgery due to longer infusions.

**Figure 1:**
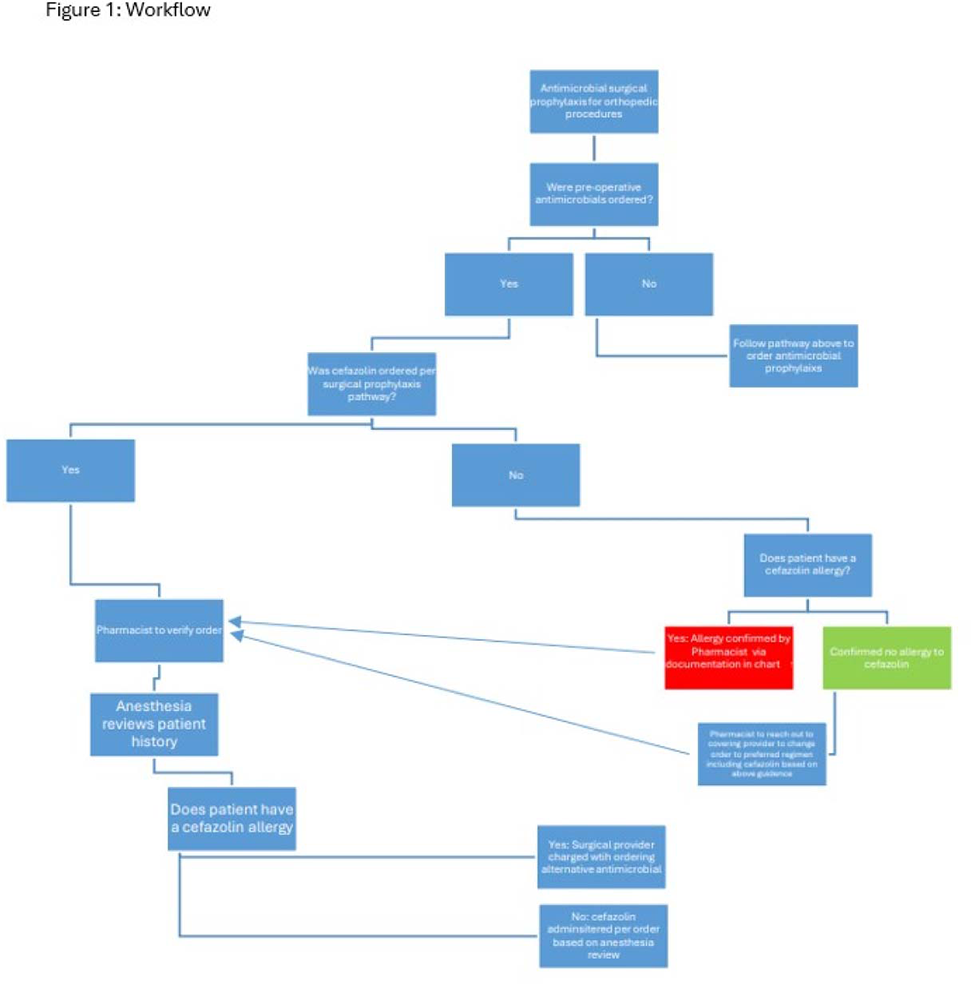
Workflow

**Figure 2:**
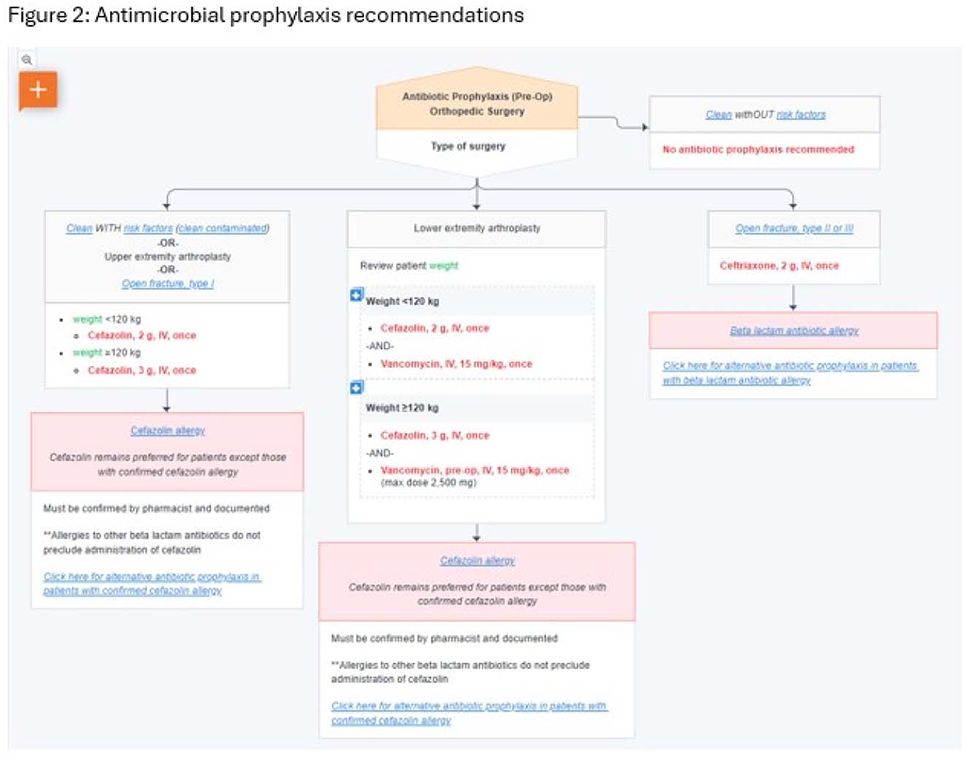
Antimicrobial prophylaxis recommendations

**Methods:**

Our team completed a multi-step evaluation including contextual inquiry to identify potential opportunities for optimization within the current workflow and collaborated with key stakeholders to identify areas for intervention.

**Table 1:**
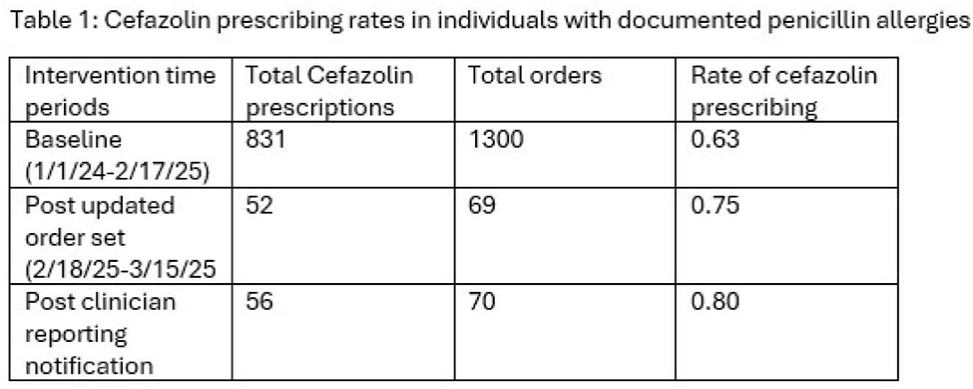
Cefazolin prescribing rates in individuals with documented penicillin allergies

**Results:**

Contextual inquiry demonstrated several key barriers including various workflows for ordering of perioperative antibiotics, identification of a CPOE alert regarding potential of cross-allergenicity, and lack of clarity for who is ultimately responsible for decision as to which antibiotic to use.

As an initial intervention, the Antimicrobial Stewardship team collaborated with orthopedics and allergy clinicians to provide guidance on risk of cross-reactivity with CFZ/common PCN-based antibiotics and the importance of updating the medical record with accurate allergy information. This educational initiative demonstrated increased CFZ prescribing which was sustained for the following two quarters, however, still left room for optimization.

Several strategies were developed and proposed to key stakeholders including integrated pathways detailing workflow and antibiotic recommendations (figures 1 and 2). After discussion, the decision was made to continue with utilization of the current order set with optimized verbiage to nudge providers to use CFZ in individuals with documented PCN allergies. In addition, individual provider ordering summaries were distributed to the individual orthopedic and anesthesiology providers detailing the rate of CFZ prescribing in these individuals as compared to their top quartile peers. Prescribing rates of CFZ increased from a baseline of 63% to 75% after optimization of the order sets and 80% post distribution of the provider summaries (table 1).

**Conclusion:**

This collaboration demonstrated the importance of engaging end users in strategy meetings as well optimizing the verbiage in CPOE systems to support recommendations.

**Disclosures:**

Amanda Binkley, PharmD, BCIDP, AAHIVP, Shionogi: Advisor/Consultant

